# Protective Effect of Clostridium butyricum on Escherichia coli-Induced Endometritis in Mice via Ameliorating Endometrial Barrier and Inhibiting Inflammatory Response

**DOI:** 10.1128/spectrum.03286-22

**Published:** 2022-11-02

**Authors:** Kexin Wang, Ke Wang, Junrong Wang, Fan Yu, Cong Ye

**Affiliations:** a Department of Anesthesiology, China-Japan Union Hospital of Jilin University, Changchun, Jilin, China; b Department of Obstetrics and Gynecology, China-Japan Union Hospital of Jilin University, Changchun, Jilin, China; c Department of Gastroenterology and Hepatology, China-Japan Union Hospital of Jilin University, Changchun, Jilin, China; Jilin University

**Keywords:** endometritis, *Clostridium butyricum*, inflammation, ZO-1, gut microbiota

## Abstract

Endometritis is a common reproductive disease occurs both in human and animals. Clostridium butyricum is a Gram-positive anaerobic bacterium that can ferment various carbohydrates into butyric acid. In this study, we investigated the effects of *C. butyricum* on Escherichia coli-induced endometritis and clarified the underlying mechanism. We first verified the protective effect of *C. butyricum in vivo* by establishing a mouse model of E. coli-induced endometritis. It was determined that *C. butyricum* pretreatment significantly reversed E. coli-induced uterine histopathological changes. Meanwhile, *C. butyricum* pretreatment significantly decreased the production of pro-inflammatory mediators and the levels of myeloperoxidase (MPO) and malondialdehyde (MDA). We found that *C. butyricum* could inhibit TLR4-mediated phosphorylation of NF-κB and the activity of histone deacetylase (HDAC). Furthermore, *C. butyricum* significantly increased the expression of the tight junction proteins (TJPs) ZO-1, claudin-3, and occludin. Additionally, treatment with *C. butyricum* culture supernatant dramatically suppressed the degree of inflammation in the uterus, and inactivated *C. butyricum* did not exert a protective effect. We subsequently investigated butyrate levels in both the uterus and blood and observed a marked augment in the *C. butyricum* treatment group. Collectively, our data suggest that *C. butyricum* maintains epithelial barrier function and suppresses inflammatory response during E. coli-induced endometritis and that the protective effect of *C. butyricum* may be related to the production of butyrate.

**IMPORTANCE** Endometritis is a common reproductive disease both in human and animals. It impairs female fertility by disrupting endometrial function. Antibiotics are widely used to treat endometritis in clinical practice, but the misuse of antibiotics often leads to antibiotic resistance. Therefore, there is an urgent need for new therapeutic agents to treat bacterial endometritis and overcome bacterial resistance. In this study, we found that C. butyricum could protect from E. coli-induced endometritis.

## INTRODUCTION

Endometritis impairs female fertility by disrupting endometrial function, generating an unfavorable environment for embryos in the female reproductive tract and interfering with follicle function and oocyte health (1, 2). Currently, endometritis represents a considerable worldwide economic problem for the animal husbandry industry, as it is highly correlated with reduced reproductive activity (3). The principal pathogenic bacterium reported to cause endometritis include Escherichia coli, Staphylococcus aureus, and Streptococcus spp. (4). During mating and birth, the endometrium is exposed to bacteria, which may lead to endometritis and eventually cause infertility. In endometritis, the uterus releases large amounts of inflammatory mediators after stimulation of neutrophils. Previous studies have shown that pro-inflammatory cytokines such as tumor necrosis factor α (TNF-α) and interleukin (IL)-1β are involved in the development of bacterial endometritis (4, 5). It has been determined that inhibition of these pro-inflammatory cytokines may reduce bacterial endometritis. To our knowledge, the epithelial barrier integrity of the endometrium is impaired and tight junction protein (TJP) content in uterine tissue is significantly reduced during endometritis (6).

Pathogenic microorganisms activate the innate immune response of the body by binding to Toll-like receptors (TLRs) and secreting inflammation-associated factors in infected sites (7). Lipopolysaccharide (LPS) is an immunostimulatory component of the cell wall in Gram-negative bacteria such as E. coli, which can activate the TLR4/NF-κB signaling pathway and cause a strong inflammatory response (8, 9). The innate immune defense system of the endometrium is composed primarily of three parts: TLRs, acute phase proteins, and antimicrobial peptides (1). Therefore, E. coli induces the production of cytokines and inflammatory mediators by activating TLRs on the endometrium, promoting the infiltration of neutrophils and macrophages and leading to endometritis (10). HADCs are a class of enzymes that remove acetyl groups from histone lysine residues, cause chromatin condensation, and reduce the expression of certain genes (11). These enzymes play an important regulatory role in anti-inflammatory functions and modulate the activity of the transcription factor NF-κB in different cell types (12, 13).

Antibiotics are widely used for the treatment of endometritis in clinical practice, but their misuse often leads to antibiotic resistance (14). Thus, there is an urgent need for new therapeutic agents to treat bacterial endometritis and overcome bacterial resistance (15). Probiotics influence the interaction between pathogenic microorganisms and the physiological functions of the host (16). Clostridium butyricum is a Gram-positive, rod-shaped, spore-forming obligate anaerobic bacterium that can ferment a variety of carbohydrates into butyric acid. It is also a common animal gut symbiotic bacterium and is frequently found in the environment. Previous studies have demonstrated that *C. butyricum* exhibits excellent protection against colitis, acute pancreatitis, bacterial vaginosis, and other inflammatory conditions (17–20). However, until recently, the protective effect of *C. butyricum* against E. coli-induced endometritis remained unknown, especially its underlying molecular mechanisms. Thus, in this trial, we evaluated the protective effect of *C. butyricum* on E. coli-induced endometritis in mice and investigated its potential mechanism of action.

## RESULTS

### *C. butyricum* alleviates histopathological changes in uterine tissues.

**FIG 1:**
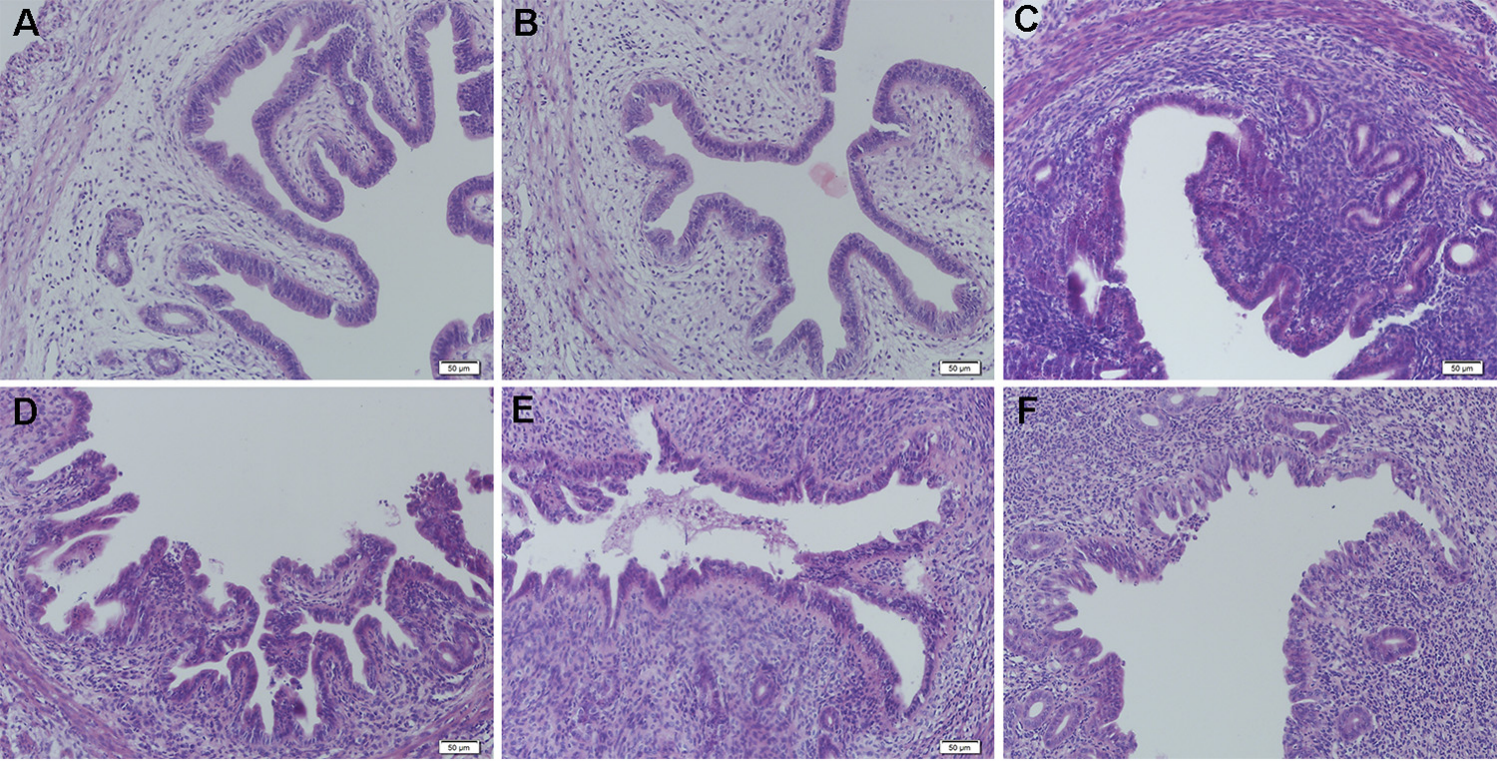
Histopathologic sections of uterine tissues (hematoxylin and eosin [H&E], ×400 magnification). (A) Control group treated with phosphate-buffered saline. (B) Group treated with Clostridium butyricum. (C) Group pretreated with Escherichia coli. (D) Group pretreated with *C. butyricum* (1 × 10^6^ CFU/mL) and E. coli. (E) Group pretreated with *C. butyricum* (1 × 10^7^ CFU/mL) and E. coli. (F) Group pretreated with *C. butyricum* (1 × 10^8^ CFU/mL) and E. coli.

The effects of *C. butyricum* on E. coli-induced histopathological changes were detected by hematoxylin and eosin (H&E) staining. As shown in Fig. 1A through F, the control group showed normal uterine tissue structure with no evident histopathological changes. Compared with the control group, the E. coli group exhibited more severe and distinct pathological changes in their uterine tissues, and diffuse infiltration of macrophages and neutrophils was observed within the endometrial glands. Furthermore, we observed stromal edema, distorted architecture, and stroma alteration in the E. coli group. However, the E. coli-induced pathological changes were markedly ameliorated by pretreatment with *C. butyricum* in a dose-dependent manner. Notably, the effect was more prominent at the highest dose of *C. butyricum* in our study, which was closely matched with the control group. In addition, compared to the damage in the E. coli group, a histological analysis showed normal morphology in the *C. butyricum* group.

### Effects of *C. butyricum* on MPO activity and MDA content.

**FIG 2:**
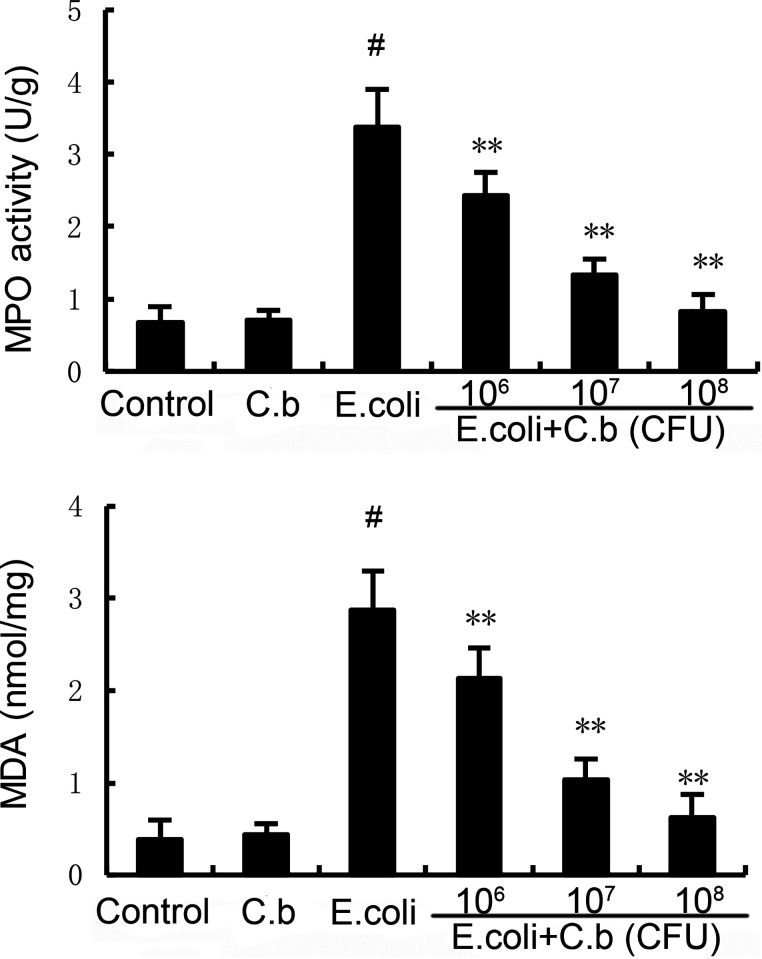
Effects of *C. butyricum* on myeloperoxidase (MPO) activity and malondialdehyde (MDA) content. Values are shown as the mean ± standard error of the mean (SEM). #, *P* < 0.01 versus control group. *, *P* < 0.05; **, *P* < 0.01 versus E. coli group.

Myeloperoxidase (MPO) is an enzyme that reflects the levels of inflammation and oxidative stress. Malondialdehyde (MDA) content is used to assess the antioxidative activity of *C. butyricum* on oxidative stress in E. coli-induced mice. In our research, E. coli stimulation led to significantly increased MPO activity and increased MDA levels in the E. coli group compared with the control group (Fig. 2). However, treatment with *C. butyricum* reduced the levels of MPO activity and MDA content compared with those in the E. coli group (Fig. 2).

### Effects of *C. butyricum* on the levels of inflammatory cytokines.

**FIG 3:**
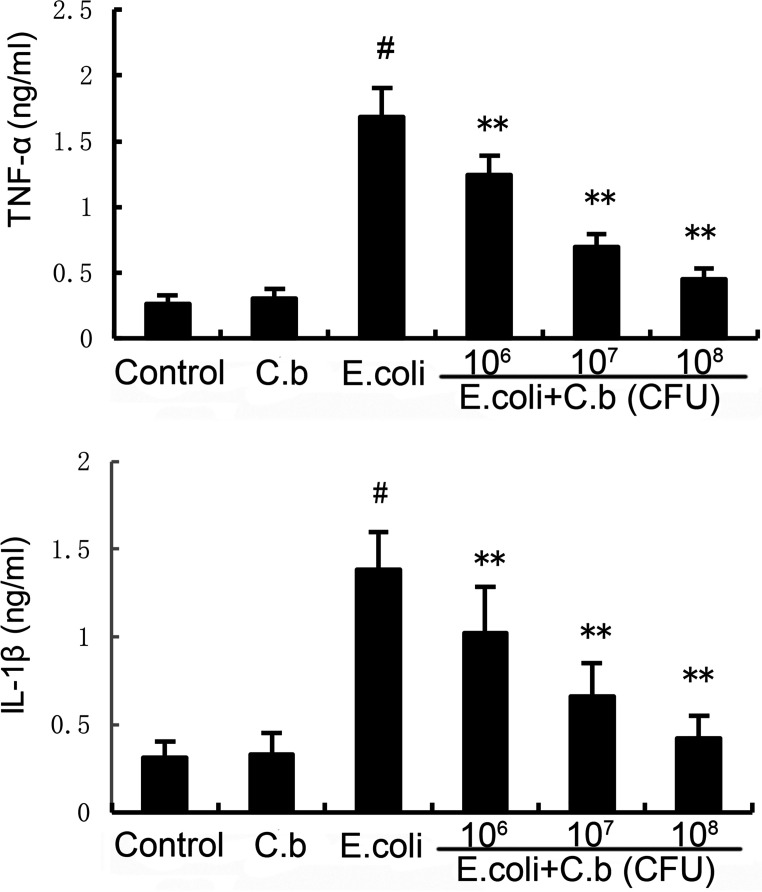
Effects of *C. butyricum* on tumor necrosis factor α (TNF-α) and interleukin (IL)-1β levels. Values are shown as the mean ± SEM. #, *P* < 0.01 versus control group. *, *P* < 0.05; **, *P* < 0.01 versus *E. coli* group.

TNF-α and IL-1β play important roles in inflammatory diseases. To evaluate the effects of *C. butyricum* on E. coli-induced endometritis, the levels of inflammatory cytokines TNF-α and IL-1β were determined by enzyme-linked immunosorbent assay (ELISA). As shown in Fig. 3, E. coli stimulation induced significant increases in TNF-α and IL-1β production relative to the control group. Compared with those in the E. coli group, the levels of TNF-α and IL-1β expression were markedly reduced in a dose-dependent manner by treatment with *C. butyricum*. In addition, there were no significant differences between the *C. butyricum* group and the control group. The results showed that *C. butyricum* decreased E. coli-induced endometritis by downregulating the proinflammatory cytokines TNF-α and IL-1β to directly inhibit the inflammatory response.

### *C. butyricum* modulates the expression of ZO-1, claudin-3 and occludin.

**FIG 4:**
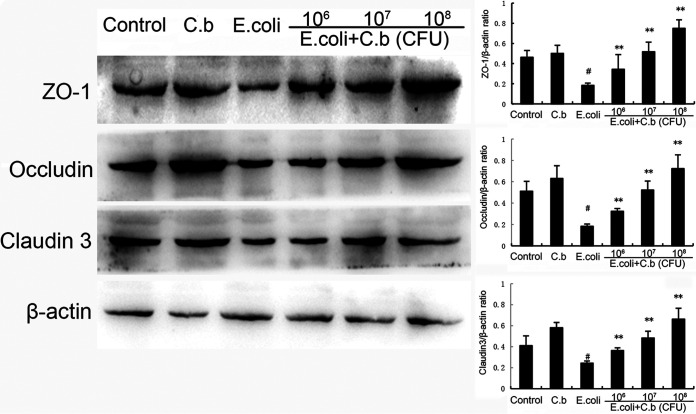
Effects of *C. butyricum* on ZO-1, claudin-3, and occludin expression. Values are shown as the mean ± SEM. #, *P* < 0.01 versus control group. *, *P* < 0.05; **, *P* < 0.01 versus E. coli group.

It has been confirmed that ZO-1, claudin-3, and occludin are important epithelial TJPs for preventing harmful substances from breaching the mucosa epithelial cells. Therefore, we furthered evaluated the effect of *C. butyricum* on TJP expression in the E. coli-induced endometritis model, and determined the levels of ZO-1, claudin-3, and occludin using Western blotting. As shown in Fig. 4, compared with levels in the control group, ZO-1, claudin-3, and occludin expression were strongly significantly inhibited by E. coli treatment. However, pre-treatment with *C. butyricum* markedly enhanced the levels of ZO-1, claudin-3, and occludin in a dose-dependent manner.

### Effect of *C. butyricum* on *E. coli*-induced TLR4/NF-κB activation.

**FIG 5:**
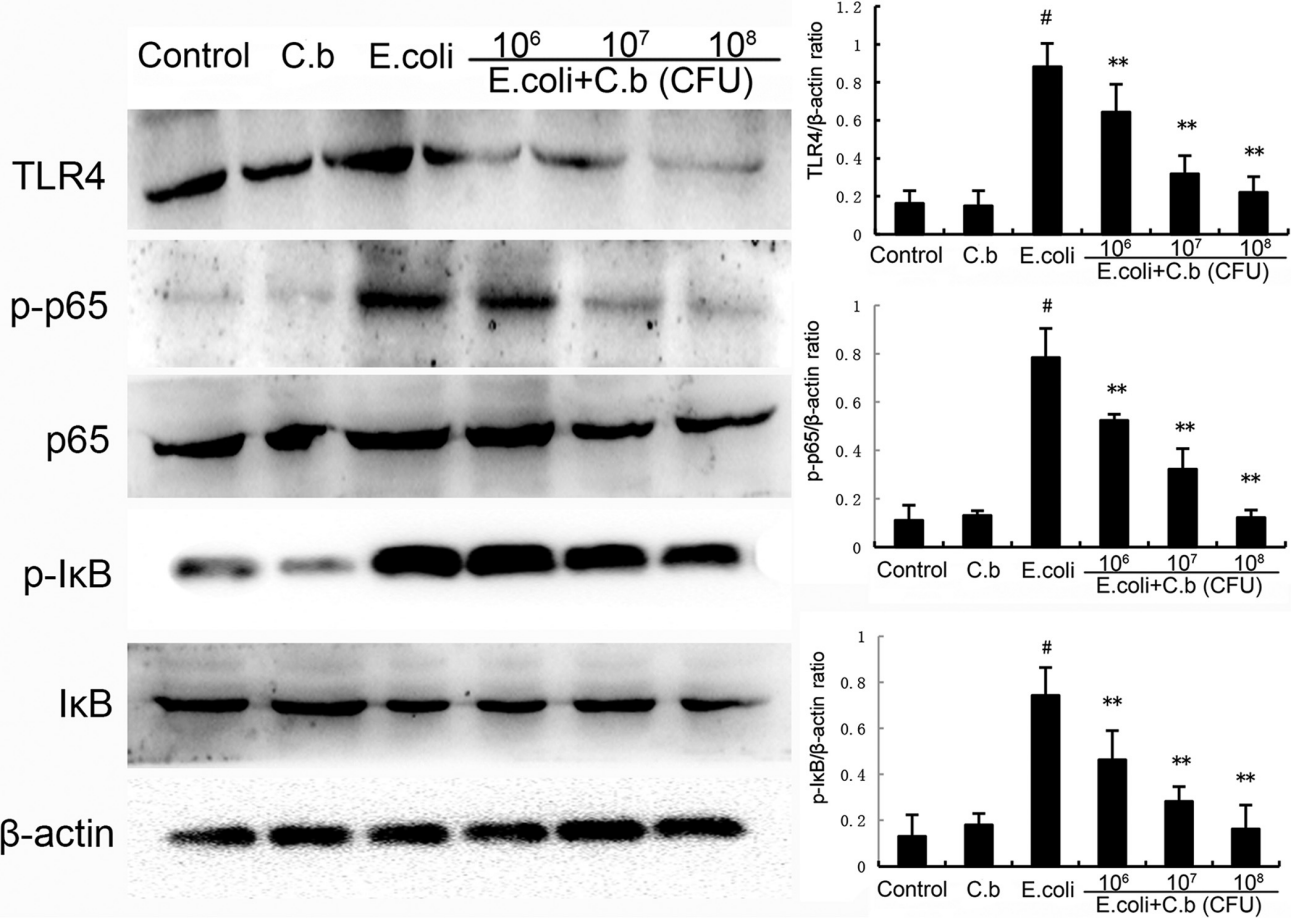
Effects of *C. butyricum* on the expression of Toll-like receptor 4 (TLR4) and NF-κB activation. Values are shown as the mean ± SEM. #, *P* < 0.01 versus control group, *, *P* < 0.05; **, *P* < 0.01 versus E. coli group.

TLR4/NF-κB plays a pivotal role in the development of inflammation. To confirm whether *C. butyricum* affects TLR4/NF-κB signaling pathway activation, we investigated the degrees of TLR4, IκB, and p65 protein expression by Western blotting. The data showed that the phosphorylation levels of NF-κB p65, and IκB and the levels of TLR4 were dramatically increased in the E. coli group compared with the control group (Fig. 5). However, these changes were markedly downregulated in the *C. butyricum* administration groups compared with the E. coli group (Fig. 5).

### Effect of *C. butyricum* on HDAC protein expression in uterine tissues.

**FIG 6:**
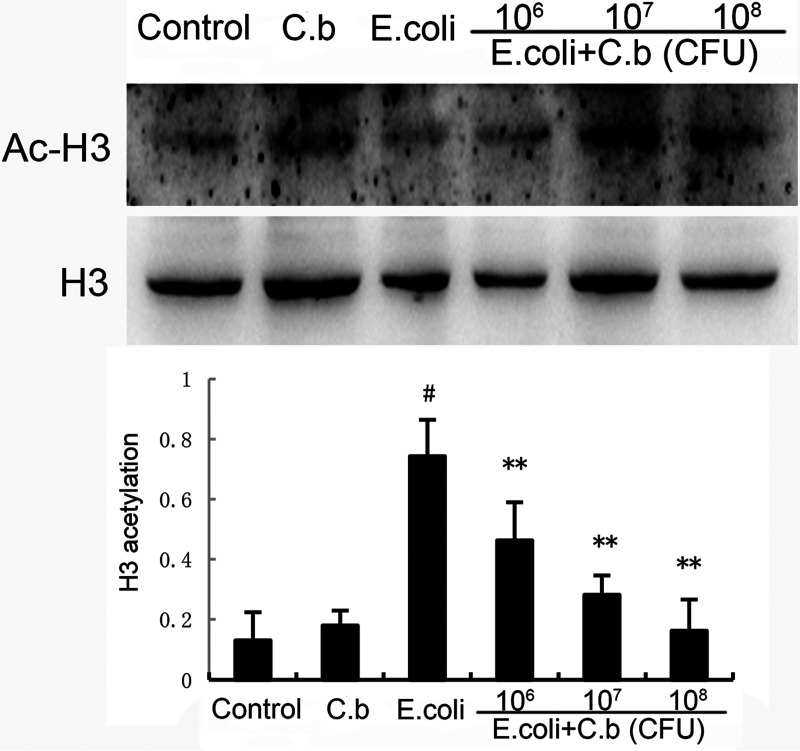
Effects of *C. butyricum* on histone deacetylase (HDAC) expression. Values are shown as the mean ± SEM. #, *P* < 0.01 versus control group. *, *P* < 0.05; **, *P* < 0.01 versus *E.coli* group.

The histone H3 acetylation (Ac-H3) levels in uterine tissues were measured by Western blotting. As shown in Fig. 6, E. coli induced the downregulation of Ac-H3 protein expression in the uterus compared to that in uterine tissues without E. coli stimulation. This effect was completely prevented by *C. butyricum* pre-treatment in a dose-dependent manner. This result indicated that *C. butyricum* protected against E. coli-induced endometritis by inhibiting HDAC.

### Effect of inactivation and SCS from *C. butyricum* on inflammatory response to endometritis.

**FIG 7:**
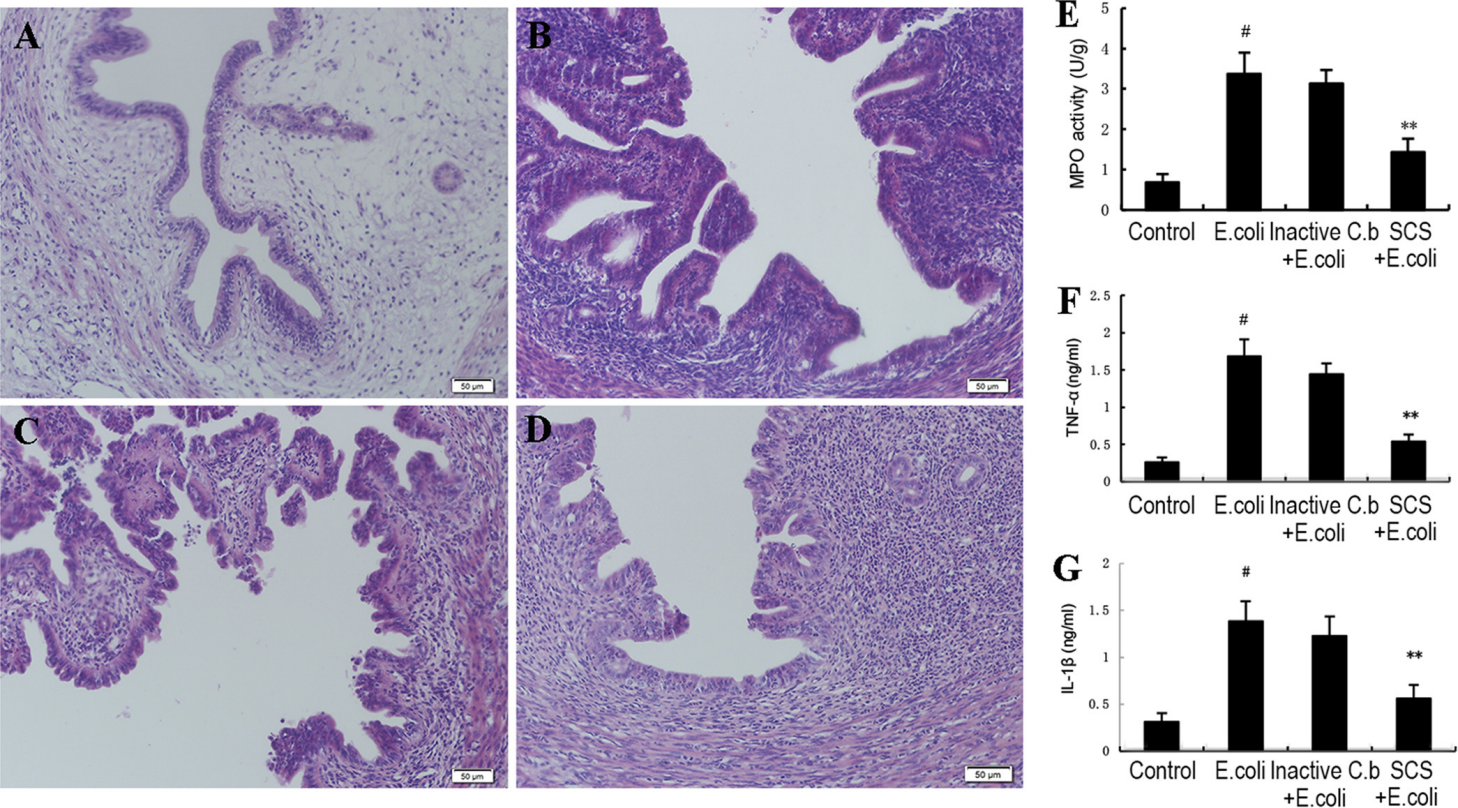
Effects of inactivation and spent culture supernatants (SCS) from *C. butyricum* on inflammatory response to endometritis. Values are shown as the mean ± SEM. #, *P* < 0.01 versus control group. *, *P* < 0.05; **, *P* < 0.01 versus *E.coli* group.

First, we tested the effects of heat-killed *C. butyricum* and spent culture supernatants (SCS) from *C. butyricum* on TNF-α and IL-1β expression in E. coli-induced endometritis. In this study, TNF-α and IL-1β levels were reduced significantly by SCS from *C. butyricum* compared with those in the E. coli-treated group (Fig. 7). Pretreatment with heat-killed *C. butyricum* did not alter TNF-α and IL-1β levels in E. coli-induced endometritis. We next evaluated the effects of inactivated *C. butyricum* and SCS from *C. butyricum* on histopathological changes in uterine tissues. In this study, pretreatment with heat-killed *C. butyricum* did not affect histopathological changes in uterine tissues. However, SCS from *C. butyricum* could inhibit E-coli-induced histopathological changes in uterine tissues (Fig. 7).

### GC-MS analyses of the blood and uterine tissues in *C. butyricum* treatment mice.

**FIG 8:**
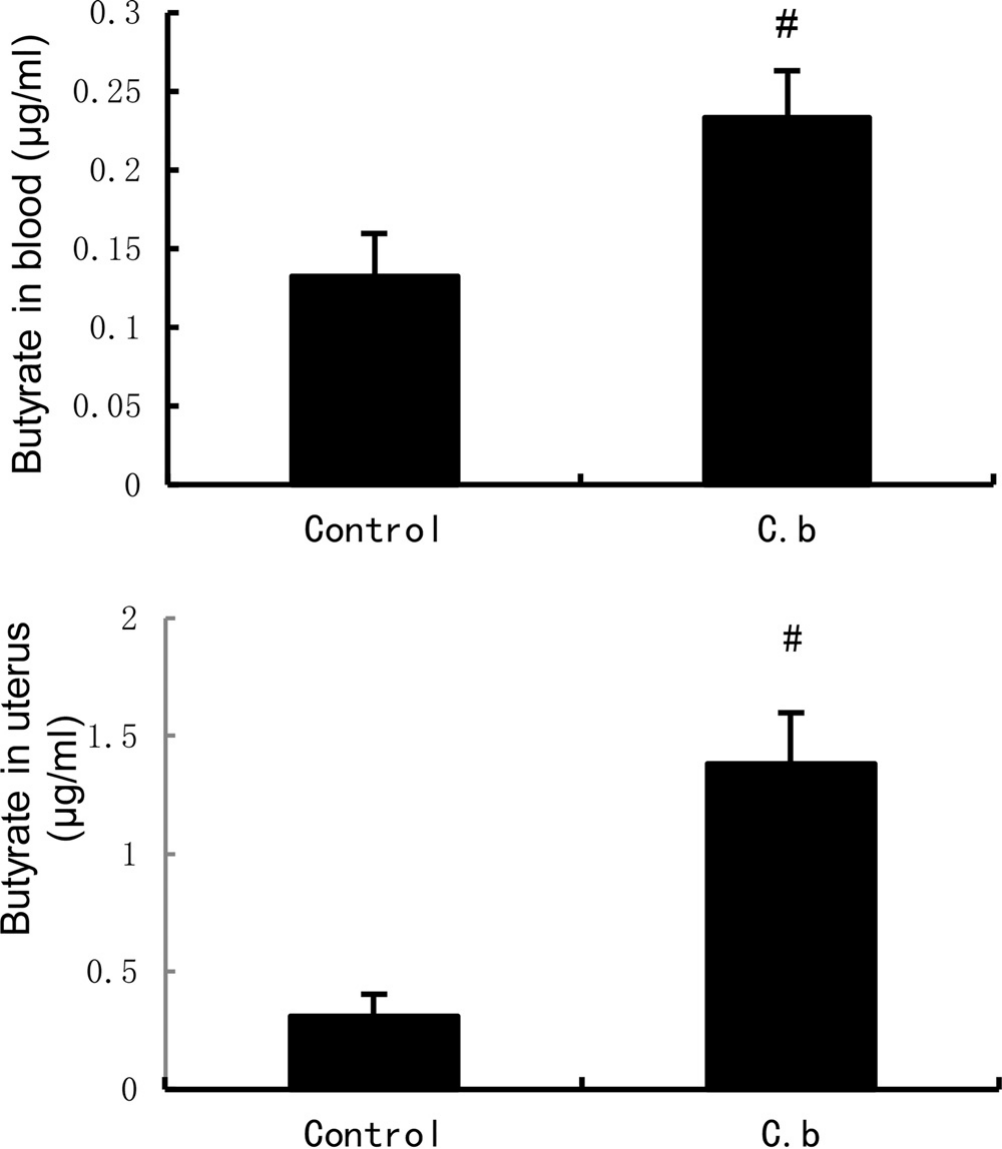
Gas chromatography-mass spectrometry analyses of the blood and uterine tissues in *C. butyricum* treatment mice. Values are shown as the mean ± SEM. #, *P* < 0.01 versus control group.

To investigate the molecular mechanism that *C. butyricum* protects against E. coli-induced endometritis in mice, we examined butyrate levels in both the blood and uterus by gas chromatography-mass spectrometry (GC-MS). As shown in Fig. 8, butyrate content in the blood and uterus was markedly elevated after pretreatment with *C. butyricum* compared with that in the control group. Thus, we concluded that butyrate was likely the active ingredient responsible for the inhibitory effect of *C. butyricum* on endometritis.

## DISCUSSION

Endometritis is considered a critical infertility element and causes enormous economic losses in the dairy industry (21). Endometritis often occurs during the postpartum period: in this period, the microbial community in the uterus fluctuates, with cycles of infection, elimination, and reinfection with bacteria (1). The bacteria most commonly isolated from animals with endometritis are E. coli, Trueperella pyogenes, and *Prevotella* and *Bacteroides* spp. (22). E. coli is the main pathogen which causes the clinical signs of uterine disease (23). Previous studies have established the endometritis model by infusing E. coli into the uteri of mice and cattle (24, 25). Therefore, we used a mouse model of E. coli-induced endometritis to investigate the effect and mechanism of endometritis prevention in cows. Meanwhile, the treatment of endometritis attracts much discussion because animals have reduced fertility even after successful treatment. In addition, endometritis not only causes pain and suffering but also perturbs ovarian function and oocyte health (1). Therefore, we suggest that prevention is better than a cure for endometritis.

Probiotics, as beneficial active microbial preparations for humans and animals, are increasingly used in the treatment of diseases and have achieved some efficacy. Our investigation revealed the protective role of *C. butyricum* on E. coli-induced endometritis in an experimental mice model. *C. butyricum* is a Gram-positive anaerobic bacterium that efficiently generates butyrate and has been shown not to produce cytotoxins (26). Additionally, it has good resistance to stomach acid and temperature (27). Several studies have demonstrated the protective effects of *C. butyricum* in colitis, immune response, intestinal barrier, etc. (17, 28, 29). Notably, many functions of gut microbiota in ameliorating disease occur via metabolites such as short-chain fatty acids (SCFAs), including acetic acid and butyric acid (30). Recently, accumulating evidence has shown that SCFAs have anti-inflammatory effects and regulate different processes, including hormone secretion, immune responses, and cell differentiation (31, 32). Previous studies have demonstrated that SCFAs have anti-inflammatory effects such as lowering NF-κB transactivation and inhibiting TNF-α, IL-6, and myeloperoxidase activity (33–35). Several studies have also shown that SCFAs may regulate the integrity of the tight junction barrier and decrease intestinal permeability and risk of injury. Butyric acid, which can be produced by fermentation of *C. butyricum* and accounts for 85% of all intestinal SCFAs (36), has been shown to inhibit TLR4-mediated signaling and reduce HDAC levels (37). This prompted us to ask whether *C. butyricum* or its metabolite, butyric acid, had similar protective effects against endometritis.

In the present study, we observed significant uterus pathological alterations, such as inflammatory cell infiltration, endometrial epithelial shedding, and serious structural damage, in the E. coli-induced endometritis group without *C. butyricum* treatment. Moreover, three different viable *C. butyricum* doses all mitigated uterus injury compared to E. coli alone. MPO activity is a marker of macrophage and neutrophil influx into parenchymal tissue (38). MPO levels significantly increased after E. coli injection compared with those in the control group. However, pretreatment with *C. butyricum* inhibited MPO activity in a dose-dependent manner in E. coli-induced endometritis mice. The MPO results also showed the anti-inflammatory activity of *C. butyricum* by suppressing the infiltration of inflammatory cells. In addition, E. coli significantly enhanced reactive oxygen species (ROS) levels in endometritis, contributing to the inflammatory reaction (39). MDA, a biomarker of oxidative stress (40), was dramatically increased in the E. coli group in our study, and *C. butyricum* significantly attenuated E. coli-induced MDA levels.

In response to E. coli treatments, the production of pro-inflammatory cytokines was clearly upregulated. Pro-inflammatory cytokines such as TNF-α and IL-1β have been found in endometritis. TNF-α is the main endogenous cytokine which stimulates the production of cellular adhesion molecules, and activation of neutrophils impairs vascular endothelial cells (41, 42). IL-1β, a subtype of IL-1, is a cytokine released early in the inflammatory response by monocytes, endothelial cells, and macrophages (43). It has been demonstrated that IL-1β is involved in the clearance of invading pathogens and stimulation of neutrophil activation (44). Consistent with results from previous studies, TNF-α and IL-1β concentrations were markedly elevated in the E. coli-induced endometritis group in this study. However, pretreatment with *C. butyricum* suppressed TNF-α and IL-1β expression in a dose-dependent manner. Therefore, we conclude that *C. butyricum* decreased inflammatory responses by reducing the release of pro-inflammatory cytokines TNF-α and IL-1β.

In this context, to further evaluate the potential molecular mechanism by which *C. butyricum* suppresses the production of pro-inflammatory factors, we hypothesized that *C. butyricum* could alleviate endometritis by suppressing the TLR4/NF-κB signaling pathway and HDAC levels. E. coli contains LPS in its cytoderm, which induces an inflammatory response by triggering TLR4-mediated signal transduction pathways, including NF-κB, mitogen-activated protein kinase, and PI3K/Akt (8, 45). As a pattern recognition receptor, TLR4 is one of the primary inflammatory signaling proteins and is crucial to activating NF-κB (46). Evidence has shown that NF-κB is an important transcription factor that plays an imperative role in inducing various pro-inflammatory chemokines and cytokines, such as IL-6, IL-1β, TNF-α, and Cox-2 (47, 48). It is a widespread heterodimeric transcription factor, and the activation of NF-κB p65 accompanies the degradation of inhibitory protein IκBα in the proteasome. Next, the activated NF-κB p65 moves into the nucleus after phosphorylation binds to specific sites and initiates the transcription of pro-inflammatory function by regulating downstream gene expression (49). As described in Fig. 5, *C. butyricum* pretreatment not only effectively inhibits IκBα degradation but also reduces NF-κB p65 gene expression. There have been few reports about augmented HDAC activity in endometritis. HDACs are related to the removal of epsilon amino groups from the lysine residue in histones, causing the agglomeration of DNA chromatin (50). Previous studies have demonstrated that inhibition of HDAC increases histone acetylation, leading to a more open structure of chromatin and promoting DNA transcription (51, 52). Among these HDACs, HDACs 1, 2, and 3 are known for their roles in promoting pathology and inflammation (53, 54). Therefore, inhibition of HDAC activity would be a novel therapeutic measure against endometritis. In our study, E. coli significantly decreased Ac-H3 levels, but pretreatment with *C. butyricum* elevated Ac-H3 levels. Our results suggest that *C. butyricum* ameliorates E. coli-induced endometritis by inhibiting the TLR4/NF-κB signaling pathway and the HDAC level.

The vulva, vagina, cervix, and cervical mucus plug supply physical barriers to fight infection during gestation (1). The next line of endometrial defense is the columnar epithelium, which has tight junctions between cells. Recent studies have shown that tight junctions play an important role in the pathogenesis of endometritis (55, 56). These structures are complexes composing of cytosolic and transmembrane proteins that restrict the diffusion of solutes through adjacent epithelial cells (56, 57). Inflammatory stimuli and barrier damage lead to downregulation of the tight junctions, which is a progression of endometrial epithelial pathology (58). Furthermore, accumulating evidence has shown that maintaining tight junction complexes can prevent uterus injury (56). ZO-1, claudin-3, and occludin proteins are key components of the intact intercellular barrier at tight junctions (59). Our reports showed that there was an obvious reduction in TJPs (ZO-1, claudin-3, and occludin) in the uterus exposed to E. coli, and treatment with *C. butyricum* had a dramatic protective effect on the TJPs.

*C. butyricum* is a vital probiotic to inhibit the growth of pathogenic species, and the possible mechanisms for its protective effect against endometritis include competition for nutrients and the production of SCFAs. In the present study, we investigated whether SCS from *C. butyricum* and heat-killed *C. butyricum* have similar protective effects against endometritis to that of activated *C. butyricum*. Also, SCS from *C. butyricum* inhibited the production of pro-inflammatory factors and activation of TLR4/NF-κB signaling pathways. However, the inactivated *C. butyricum* was not significantly discrepant with the E. coli group. These results suggest that the anti-inflammatory effects of *C. butyricum* may be due to the production of SCFAs, such as butyric acid and propionic acid. Interestingly, we found that both uterus and blood butyric acid levels were significantly increased after *C. butyricum* treatment compared to those in the control group. Other reports have definitively shown that butyrate, as a function of HDAC inhibition, has anti-inflammatory effects by lowering NF-κB transactivation and upregulating TJP expression (60–63). Therefore, our results are consistent with those from previous studies. For these reasons, we propose butyrate as a treatment to ameliorate E. coli-induced endometritis.

In conclusion, these data suggest that *C. butyricum* exerted a protective effect against E. coli-induced endometritis in mice. These effects include suppression of inflammatory cytokine production, inhibition of ROS production and neutrophil infiltration, and activation of endometrial protection barriers. In particular, we elucidate the molecular mechanisms for the beneficial effects of *C. butyricum*, which downregulates TLR4/NF-κB signal pathways and decreases HDAC activity. Furthermore, the study suggests that butyric acid, a metabolite of *C. butyricum*, may be involved in the protection of *C. butyricum* against endometritis induced by E. coli treatment. Therefore, *C. butyricum* and its metabolite butyric acid could be prospective therapeutic agents for the prevention and management of endometritis in dairy cattle. Based on these encouraging results in mice, further investigations should be carried out to evaluate the protective effects of *C. butyricum* against endometritis in cattle.

## MATERIALS AND METHODS

### Ethics statement.

All animal procedures were performed in strict accordance with the legislation for care and use of laboratory animals of the People’s Republic of China and approved by the Animal Ethics Committee of Jilin University.

### Materials.

Escherichia coli O111:K58 (CVCC1450) was obtained from the China Institute of Veterinary Drug Center (Beijing, China) and cultured in nutrient Luria-Bertani (LB) broth (Sigma-Aldrich L3022, Burlington, MA, USA) overnight at 37°C with shaking. Clostridium butyricum (ATCC 19398) was obtained from the China General Microbiological Culture Collection Center and cultured in sterile Reinforced Clostridial Medium at 37°C under anaerobic conditions until the logarithmic phase of growth with a bacterial density of 0.5 at *A*_600_. Primary antibodies for ZO-1, occludin, claudin 3, TLR4, β-actin, and phosphorylated and nonphosphorylated forms of NF-κB were purchased from Cell Signaling Technologies, Inc. (Beverly, MA, USA). Horseradish peroxidase (HRP) secondary antibody and the primary antibody of H3, Ac-H3, were purchased from GeneTex (Irvine, CA, USA).

### Animals.

Specific pathogen-free, female BALB/c mice (6 to 8 weeks old, 22 to 25 g) were provided from the Center of Experimental Animals of Baiqiuen Medical College of Jilin University (Jilin, China). The animals were provided with 11 to 13 h light and dark cycles under specific pathogen-free conditions with a room temperature of approximately 25 ± 5°C. Mice were raised for 1 week with free access to water and food to adapt to the feeding environment before the experiment.

### Preparation of inactivation and the SCS of *C. butyricum*.

The inactivation and SCS of *C. butyricum* were performed according to a modified method (29, 64, 65). In summary, the culture broth of *C. butyricum* was centrifuged at 12,000 rpm for 10 min. Then, the bacteria were suspended in sterile phosphate-buffered saline (PBS) and heat-killed at 121°C for 30 min. Next, *C. butyricum* was collected, washed 3 times with PBS, and suspended in sterile saline at a final concentration of 1 × 10^8^ CFU/mL for subsequent treatments. SCS from *C. butyricum* were obtained by centrifugation of the bacterial solution at 8,000 × *g* for 30 min and then filtrated through a sterile filter with a pore size of 0.22 μm and stored at low temperature.

### Mouse uterine endometritis model and experimental design.

All-female BALB/c mice were stochastically divided into the following 6 experimental groups, with 8 mice in each group. (i) The control group was composed of healthy mice that did not receive any treatment. (ii) For the E. coli group, inocula containing E. coli 1 × 10^5^ CFU/mL were prepared with sterile normal saline before the experiment. Mice then received a 50-μL injection of E. coli into each side of the uterus horn and no drug treatment. (iii) For the *C. butyricum* treatment groups, E. coli-infected mice received an intragastric dose of *C. butyricum* with different contents: low- (1 × 10^6^ CFU/mL), medium- (1 × 10^7^ CFU/mL), and high-doses (1 × 10^8^ CFU/mL). Mice were then treated with 200 μL *C. butyricum* once a day for 1 week before E. coli infection. (iv) For the individual *C. butyricum* group, mice received a 1-week oral administration of *C. butyricum* (1 × 10^8^ CFU/mL) and no other treatment.

To investigate the mechanism by which *C. butyricum* plays a protective role in endometritis, the female mice were divided into 4 groups (*n* = 8) at random: control group, E. coli group, *C. butyricum* SCS (200 μL of supernatant from 1 × 10^8^ CFU *C. butyricum*/day) + E. coli group, and inactivated *C. butyricum* (1 × 10^8^ CFU at 200 μL/day) + E. coli group. The mice were euthanized with sodium pentobarbital after 24 h of treatment with E. coli and the uterine samples were collected and stored at −80°C until analysis.

### Histological assay of the uterine tissues.

After the mice were sacrificed, the uterus tissues were gathered and washed 2 to 3 times with PBS. After immersed in 4% paraformaldehyde solution for 24 h, the tissues were embedded in paraffin, cut into 5-μm thick sections, and later stained with hematoxylin and eosin (H&E) reagent. After staining, the pathological tissue sections were subsequently observed under an optical microscope (Olympus, Japan). Photos were taken after typical spots had been found.

### MPO and MDA evaluation.

Uterus tissues were homogenized with reaction buffer (weight:volume ratio of 1:19) using a glass homogenizer and centrifuged at 13,000 rpm to collect the supernatant. The MPO activity and MDA content in samples was determined using commercial kits (Nanjing Jiancheng Bioengineering Institute, Nanjing, China). The absorbance peak (optical density, OD) was determined at a wavelength of 460 nm by a microplate reader and defined as MPO activity = (ODtest − ODcontrol)/(11.3 × weight [g]). MDA content in uterine tissues was measured according to the manufacturer’s instructions.

### Cytokine index of uterus tissues.

The effects of *C. butyricum* on the levels of cytokines in uterus tissues were measured by ELISA. Briefly, uterine tissues were collected from each group, homogenized in PBS on ice, and then centrifuged at 12,000 rpm for 10 min. The levels of TNF-α and IL-1β in the supernatants were determined by ELISA (BioLegend, San Diego, CA, USA) according to the manufacturer’s directions. The absorbance was measured at 450 nm using a microplate reader and the concentrations of TNF-α and IL-1β were determined using the standard curve generated from the known values.

### Western blot analysis.

Samples of uterine tissues were collected, and the total proteins were extracted by a total protein extraction reagent (Thermo Fisher Scientific, Waltham, MA, USA) according to the manufacturer’s recommended protocol. The concentration of the protein was measured with a bicinchoninic acid protein test kit (Thermo Fisher Scientific). Next, protein extracts were separated by sodium dodecyl sulfate-polyacrylamide gel electrophoresis (SDS-PAGE) and transferred to polyvinylidene difluoride (PVDF) membranes. The membranes were blocked with 5% skimmed milk in Tris-buffered saline with Tween 20 (TBST) for 3 h at room temperature. Subsequently, the membranes were treated with specific primary antibodies (1:1,000 to 1:2,000 dilutions) at 4°C overnight, washed 3 times for 15 min each with TBST and secondary HRP-conjugated anti-mouse (1:10,000) or anti-rabbit (1:5,000) IgG antibodies and incubated at room temperature for 2 h. After 3 washes times with TBST, the blots were detected using an enhanced chemiluminescence reagent kit (Beyotime, China) and visualized with the Bio-Rad imaging system (Bio-Rad, Hercules, CA, USA). All data are presented as the ratio of the target protein to the internal control (β-actin).

### Butyric acid of blood and uterus determination by GC-MS.

At the end of the 7-day-experimental period (1 × 10^8^ CFU *C. butyricum*/mL), mice were euthanized, and uterus and blood samples were collected. Blood was kept on ice for 30 min and centrifuged at 3,500 rpm for 10 min at 4°C, and serum was obtained for analysis. The samples obtained for measurement of butyric acid (a short-chain fatty acid) were immediately frozen at −20°C and stored at −80°C until quantification. Butyric acid measurement was determined following a modified protocol, as previously described. First, a standard curve sample was prepared. Next, the SCFA-containing ether layers were obtained and pooled for GC-MS analysis using an Agilent 7890 gas chromatography system coupled with an Agilent 7000D mass spectrometer. The mass spectrometry data were acquired in MRM mode with a solvent delay of 3 min.

### Statistical analysis.

All values are presented as the mean ± standard error of the mean. The images were generated by using GraphPad Prism 8.0 software (La Jolla, CA, USA) and data analysis was performed using SPSS 20.0 (SPSS Inc., Chicago, IL). Each group included three technical or biological replicates, and differences between the mean values of normally distributed data were measured by one-way analysis of variance followed by Tukey’s multiple-comparisons test. The results were considered statistically significant at *P* < 0.05 and very significant at *P* < 0.01.

### Data availability.

The raw data supporting the conclusion of this article will be made available by the authors, without undue reservation, to any qualified researcher.
